# Transmission of Secondary Syphilis

**Published:** 1860-09

**Authors:** P. Diday

**Affiliations:** Lyons


### TRANSMISSION OF SECONDARY SYPHILIS.

To the Editor of the London Lancet.

Sir,—The reviewer of Mr. Harrison's book on Venereal Diseases, mentions the author's opinions respecting the transmission of secondary syphilis. Mr. Harrison thinks that this disease, which is supposed to be directly conveyed from the male to the female, does, in reality, reach the latter very seldom otherwise than by the intermediate action of the fœtus contaminated by the father.

I am certainly extremely happy to be supported by Mr. Harrison's authority, but I must beg to observe that not only have I long ago expressed this opinion, but also based it upon the very arguments that Mr. Harrison brings forward. The author, for instance, says—

1st. That in most of the cases of ascertained secondary transmission the disease has been conveyed from the male to the female, and not from the female to the male.

2ndly. That in such cases the first symptoms which are observed upon the woman appear in regions which leave no room for suspecting infection by coitus or any other contact.

3rdly. That the female then presents neither primary chancre nor bubo.

I repeat that I am very glad to see my arguments sanctioned by the approbation of the learned author, who doubtless has clinically verified their value. Bnt I must remind your readers that these arguments have been put forward by myself, pretty well in the same words as used by Mr. Harrison, in my work entitled "On the New Doctrines of Syphilis," (Paris 1858.)

P. DIDAY.

Lyons, 1860.

